# Glomus Tumor Presenting as Raynaud's Phenomenon

**DOI:** 10.1155/2012/380540

**Published:** 2012-06-28

**Authors:** M. H. Abdelrahman, M. Hammoudeh

**Affiliations:** Rheumatology Section, Department of Medicine, Hamad General Hospital, P.O. Box 3050, Doha, Qatar

## Abstract

Glomus tumors are rare tumors that often include hands and feet; they present characteristically with paroxysmal pain, exquisite point tenderness, and cold sensitivity. Such diagnosis needs to be confirmed by imaging like ultrasound and magnetic resonance imaging (MRI). Surgical excision is the treatment of choice for glomus tumors. There are only few case reports of glomus tumors in association with Raynaud's phenomenon; this is considered to be the 4th case.

## 1. Case Report

**Figure 1:**
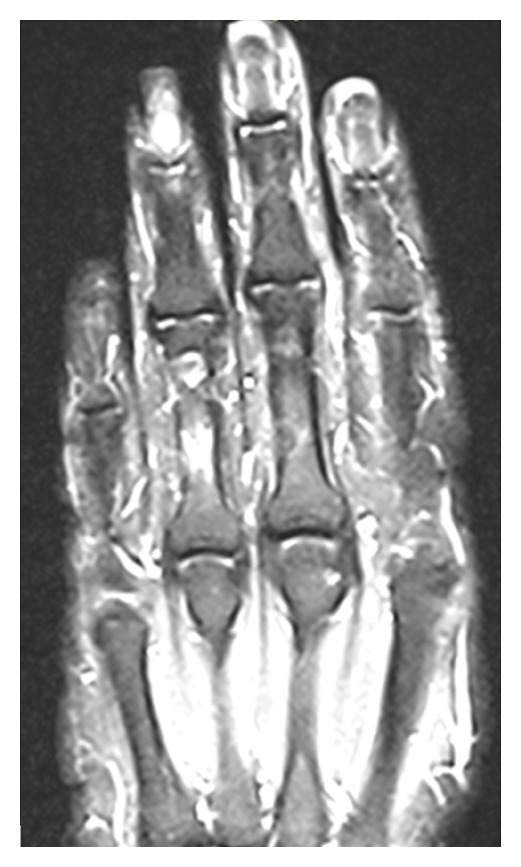
MRI of right hand showing glomus tumor of 4th digit.

43-year-old female, previously healthy, presented with episodic severe pain affecting her right 4th finger for many years. The involved area is specific to the extent that the patient can pinpoint the exact area. The condition provoked by pressing on that area and by cold exposure. The attack manifests with pain followed by a color change of the finger (blue then red), only at the distal phalanx, and attacks last about 10 to 20 minutes. She is a housewife with no history of trauma or paraesthesia. She is not on long-term medications and has no symptoms suggestive of connective tissue disease. On examination, skin examination was normal with no localized tenderness or swelling. Tinnel and phalen tests were negative. MRI (Figure 1) showed a small lesion measuring 0.6 cm × 0.6 cm subjacent to the nail bed of the fourth finger. On T1-weighted images, the lesion appears of low signal. On T2 sequences, the lesion appears of bright signal. After i.v. gadolinium administration, the lesion appears intensely enhanced. The MRI findings were going with the diagnosis of glomus tumor. The patient referred to a hand surgeon and surgical excision, and histopathology confirmed glomus tumor. Patient recovered well, and, within 10 days, pain subsided, and Raynaud's phenomenon resolved.

## 2. Discussion

Glomus tumors were described by Wood in 1812, but it is not until 1924 that Masson coined the name (glomus tumor). These are rare tumors, making, at most, 5% of tumors of the hand. The hands are the most frequent site involved in women. In men, the tumor occurs more frequently at other sites. Usually, it affects patients in middle age, but cases are described in all age groups. They are benign tumors derived from structures known as glomus bodies [1]. Glomus tumors are reported almost everywhere, and they are reported to be associated with neurologic conditions, like carpal tunnel syndrome [2]. 

Raynaud's phenomenon reported in the weirdest places. It is reported to occur in the lungs [4] and the tongue [5]. It was also well documented to occur in the nipples and responds to treatment with nifedipine [6]. It can occur in the penis, and it is a known cause of erectile dysfunction [7].

Not only that, even central nervous system involvement [3] has been shown in patients with Raynaud's.

There are few case reports of glomus tumors and Raynaud's phenomenon [8–10]. In this case, the patient had features suggestive of Raynaud's phenomenon (distal phalanges changes to blue and red with the attacks) that involve the distal phalanx of one finger. In conclusion, glomus tumors should be considered among the causes of Raynaud's phenomenon, especially that the treatment in this case is only surgical, and it is curative.
